# Bayesian Networks and Causal Discovery

**DOI:** 10.3390/e28040438

**Published:** 2026-04-13

**Authors:** Xiaoguang Gao, Zidong Wang

**Affiliations:** 1 School of Electronics and Information, Northwestern Polytechnical University, Xi’an 710129, China; 2 Department of Computer Science, City University of Hong Kong, Hong Kong SAR, China

The discovery of the precise causal representations underlying complex data forms the bedrock of artificial intelligence research. Moving beyond mere statistical correlation to understand true causal mechanisms is essential for building robust, interpretable, and adaptable intelligent systems [1]. As a foundational probabilistic graphical model, a Bayesian Network (BN) utilizes a directed acyclic graph (DAG) in which nodes represent random variables and directed edges represent conditional dependencies [2,3,4]. These relationships, quantified by a set of conditional probability distributions, provide a rigorous mathematical framework for reasoning under uncertainty [5]. Over recent years, BNs and causal discovery algorithms have been successfully applied to explore causality in a wide array of complex fields, ranging from fault detection and reliability analysis to autonomous navigation and medical diagnosis, which are increasingly supported by advanced local search strategies.

Despite these significant advancements, several critical gaps in knowledge remain. Traditional causal discovery methods often struggle when faced with high levels of non-Gaussian noise, unobserved confounding factors, or severe environmental degradations. Furthermore, while the rise of continuous optimization algorithms and deep learning has revolutionized structure learning and predictive modeling, these architectures frequently require careful regularization to maintain the interpretability required for high-stakes decision-making. Effectively combining Bayesian principles with neural networks—such as through deep Bayesian learning, information-theoretic learning, or graph neural networks—to maintain both high predictive accuracy and causal interpretability remains an ongoing challenge. There is a pressing need for novel causal models that can systematically integrate expert intuition and prior knowledge with real-time data, robustly representing causality under complex, real-world constraints [6].

This Special Issue, “Bayesian Networks and Causal Discovery,” was organized to provide a forum for researchers to address these exact challenges. It comprises eight original contributions that span theoretical advancements, algorithmic innovations, and practical applications, collectively pushing the boundaries of what causal and probabilistic models can achieve.

A significant portion of the contributions focuses on enhancing the robustness of Bayesian and neural models against severe noise and uncertainty. Hong and Kuruoglu [7] address the robustness of BNNs by proposing a Minimax Bayesian Neural Network framework. By formulating a two-player game between a deterministic neural network and a sampling stochastic neural network, their method introduces a conservative variant of a BNN that effectively adjusts variance levels and improves resilience against noise perturbations. Similarly tackling non-Gaussian impulsive noise, Xie et al. [8] introduce MEE-LSTM, a robust trajectory prediction framework for mobile robots. By replacing the traditional Mean Squared Error objective with the Minimum Error Entropy (MEE) criterion, their model utilizes an intrinsic gradient clipping mechanism to suppress outliers, achieving highly reliable causal inference in degraded sensing environments.

Another major theme of this Special Issue is the integration of expert knowledge with data-driven structure learning. Zeng et al. [9] develop the Tree-Hillclimb Search method for naval air defense threat assessment. Their approach uses an initial network structure constructed from expert knowledge as a hard constraint during structure learning, successfully balancing model accuracy with computational efficiency and interpretability. In a related vein, Zhong et al. [10] present a unified BN-based Multicriteria Decision-Making (MCDM) framework for evaluating Unmanned Aerial Vehicle (UAV) swarm performance. By employing variance decomposition, their framework establishes a bidirectional mapping between expert-assigned weights and the network’s probabilistic parameters, creating a seamless fusion of subjective expertise and objective operational data.

This Special Issue also heavily features the challenge of causal discovery in complex spatiotemporal and physical systems. Zhang et al. [11] present CRAFormer, a causal role-aware Transformer for landslide displacement forecasting. Their method learns a dynamic-lag causal graph (DLCG) from historical observations to partition drivers into distinct causal roles, applying non-anticipative masks that preserve clear causal semantics without relying on brittle signal decomposition. Addressing spatiotemporal tracking, Ma et al. [12] introduce a Probabilistic Multiple Hypothesis Tracking algorithm based on Belief Propagation (PMHT-BP). By constructing a data association factor graph and utilizing minimum spanning tree clustering, their method significantly improves the joint estimation of kinematic states for multiple resolvable group targets during complex splitting and merging events.

Finally, several articles highlight the integration of Bayesian-inspired mechanisms into computer vision architectures to handle extreme environmental ambiguity. Di et al. [13] develop GAME-YOLO for the detection of low-altitude, slow-speed, small UAVs in low-visibility scenes. By embedding a visibility restoration front-end and adaptive multi-scale fusion, their Bayesian-inspired probabilistic reasoning framework effectively manages aleatoric uncertainty. In a parallel study, Di et al. [14] introduce MFE-YOLO for Printed Circuit Board (PCB) defect detection. By reinterpreting the Convolutional Block Attention Module through a Bayesian lens as a feature-wise uncertainty weighting mechanism and redesigning the FIoU loss function, their model implicitly captures localization uncertainty, significantly reducing false alarms in automated manufacturing.

Looking forward, the research presented in this Special Issue underscores several primary avenues for future investigation. First, the integration of causal discovery with large-scale foundation models and Large Language Models (LLMs) represents a highly promising frontier. Future research must focus on scaling these algorithms to handle massive, high-dimensional datasets without sacrificing the rigorous mathematical guarantees of traditional BNs. Second, developing causal models that are inherently robust to distribution shifts and unmeasured confounders will be essential for deploying these systems in safety-critical applications like autonomous driving and personalized medicine. Finally, bridging the gap between purely data-driven continuous optimization and domain-knowledge-driven causal inference will remain a central focus for the artificial intelligence community [15], ensuring that future systems are not only highly accurate but also transparent, accountable, and fundamentally understandable.

In conclusion, the articles in this Special Issue fittingly showcase the diversity of topics embraced by Bayesian networks and causal discovery, while also suggesting timely and vital lines of research for the future.

## References

[1] Pearl J. (2009). Causality: Models, Reasoning, and Inference.

[2] Koller D., Friedman N. (2009). Probabilistic Graphical Models: Principles and Techniques.

[3] Peters J., Janzing D., Schölkopf B. (2017). Elements of Causal Inference: Foundations and Learning Algorithms.

[4] Vowels M.J., Camgöz N.C., Bowden R. (2023). D’ya Like DAGs? A Survey on Structure Learning and Causal Discovery. ACM Comput. Surv..

[5] Glymour C., Zhang K., Spirtes P. (2019). Review of Causal Discovery Methods Based on Graphical Models. Front. Genet..

[6] Schölkopf B., Locatello F., Bauer S., Ke N.R., Kaltenpoth D., Goyal A. (2021). Toward Causal Representation Learning. Proc. IEEE.

[7] Hong J., Kuruoglu E.E. (2025). Minimax Bayesian Neural Networks. Entropy.

[8] Xie D., Li Z., Zhang C., Wang C., Wei X. (2026). Robust Trajectory Prediction for Mobile Robots via Minimum Error Entropy Criterion and Adaptive LSTM Networks. Entropy.

[9] Zeng Z., Peng J., Feng Q. (2025). Tree–Hillclimb Search: An Efficient and Interpretable Threat Assessment Method for Uncertain Battlefield Environments. Entropy.

[10] Zhong R., Wang Z., Wang H., Jin Y., Bai S., Gao X. (2025). Bridging Intuition and Data: A Unified Bayesian Framework for Optimizing Unmanned Aerial Vehicle Swarm Performance. Entropy.

[11] Zhang F., Ji Y., Liu X., Liu S., Lu Z., Sun X., Ren S., Jia X. (2026). Bayesian-Inspired Dynamic-Lag Causal Graphs and Role-Aware Transformers for Landslide Displacement Forecasting. Entropy.

[12] Ma T., Shi P., Liu S., Wang P. (2026). A Novel Belief Propagation-Based Probabilistic Multiple Hypothesis Tracking Algorithm for Multiple Resolvable Group Targets. Entropy.

[13] Di R., Fan H., Ma Y., Wang J., Qian R. (2025). GAME-YOLO: Global Attention and Multi-Scale Enhancement for Low-Visibility UAV Detection with Sub-Pixel Localization. Entropy.

[14] Di R., Fan H., Feng H., Lv Z., Shu L., Xie R., Qian R. (2026). MFE-YOLO: A Multi-Scale Feature Enhanced Network for PCB Defect Detection with Cross-Group Attention and FIoU Loss. Entropy.

[15] Pearl J. (2019). The Seven Tools of Causal Inference, with Reflections on Machine Learning. Commun. ACM.

